# Buruli-RifDACC: Evaluation of the efficacy and cost-effectiveness of high-dose versus standard-dose rifampicin on outcomes in *Mycobacterium* *ulcerans *disease, a protocol for a randomised controlled trial in Ghana

**DOI:** 10.3310/nihropenres.13332.1

**Published:** 2022-11-29

**Authors:** Yaw Ampem Amoako, Abigail Agbanyo, Jacob Novignon, Lucy Owusu, Joseph Tuffour, Adwoa Asante-Poku, Yohannes Hailemichael, Iris Mosweu, Ruth Canter, Charles Opondo, Elizabeth Allen, Catherine Pitt, Dorothy Yeboah-Manu, Stephen L. Walker, Michael Marks, Richard Odame Phillips, SHARP collaboration

**Affiliations:** 1 Kumasi Centre for Collaborative Research, Kumasi, Ghana; 2 School of Medicine and Dentistry, Kwame Nkrumah University of Science and Technology, Kumasi, Ghana; 3 Department of Economics, Kwame Nkrumah University of Science and Technology, Kumasi, Ghana; 4 Noguchi Memorial Institute for Medical Research, University of Ghana, Accra, Ghana; 5 Armauer Hansen Research Institute, Addis Ababa, Ethiopia; 6 London School of Hygiene and Tropical Medicine, London, UK

**Keywords:** Buruli ulcer, Randomised Controlled Trial, High Dose Rifampicin, DACC, cost-effectiveness

## Abstract

**Background:**

Buruli ulcer (BU) can lead to disfiguring ulcers and permanent disability. The 2030 World Health Organization (WHO) road map for Neglected Tropical Diseases (NTDs) calls for major scaling up in diagnosis and management to eliminate disability due to the disease. Current treatment for BU is with daily oral rifampicin (10mg/kg dose) and clarithromycin (15mg/kg dose) for eight weeks, combined with standard gauze wound dressings. Dialkylcarbamoyl chloride (DACC)-coated dressings have been shown to irreversibly bind bacteria on wound surfaces resulting in their removal when dressings are changed. This trial aims to determine whether combining a high-dose oral rifampicin regimen with DACC dressings can improve the rate of wound healing relative to standard-dose oral rifampicin combined with DACC dressings.

**Methods:**

This is an individual, multi-centre Phase 3 randomised controlled trial, which will be conducted in three clinical sites in Ghana. The primary outcome measure will be the mean time to clearance of viable mycobacteria. Cost and health-related quality of life data will be collected, and a cost-effectiveness analysis will be performed.

**Discussion:**

The findings from this trial could lead to a change in how BU is treated. A shorter but more efficacious regimen would lead to improved treatment outcomes and potentially substantial financial and economic savings.

**Trial registration:**

Pan African Clinical Trials Repository (registration number; PACTR202011867644311). Registered on 30 ^th^ November 2020.

## Plain English summary

Buruli ulcer (BU), caused by *Mycobacterium ulcerans*, manifests clinically as a wound or swelling. There are several approaches for managing this condition. One is the availability of two antibiotics, usually rifampicin in combination with clarithromycin, that can be used to treat the disease. Rifampicin is thought to be the most important of these two drugs. Scientists have found out that a higher dose of rifampicin is safe and may help improve healing outcome and shorten the duration of treatment.

Individuals with BU wounds also go through wound dressing procedures at their hospitals and health centres. Commonly, wounds are dressed using Vaseline gauze and bandages. However, it has been observed that some affected individuals heal faster than others even with the antibiotic treatment. Some still have living organisms in their wounds many weeks after the antibiotic treatment. There is a new dressing material called DACC which is believed to permanently bind bacteria on the wound surface leading to their removal when the dressings are changed. This may be a good way to treat and prevent infection without the use of more drugs.

This study aims to determine whether combining a high-dose oral rifampicin regimen with DACC dressings can improve the rate of wound healing relative to standard-dose oral rifampicin combined with DACC dressings. Furthermore, cost and health-related quality of life data will be collected and a cost-effectiveness analysis will be performed.

The findings from this trial could lead to a change in how BU is treated. A shorter but more efficacious regimen would lead to improved treatment outcomes and potentially substantial financial and economic savings.

## Introduction

Buruli ulcer (BU), a neglected tropical disease (NTD) caused by infection with *Mycobacterium ulcerans* (MU), occurs in more than 30 countries worldwide with a focus in rural West Africa ^1^. In Ghana, the disease is confined to the south, and in particular parts of the Ashanti Region ^2^. Access to treatment in rural areas can be challenging and late presentation is typical, due to fear, stigma, suspicion about conventional medicine and high cost of care-seeking for affected individuals and their families ^3, 4^. The mode of transmission remains unknown; however, children aged five to 15 years are most commonly affected ^5^. Initially, the disease manifests as either a subcutaneous painless nodule tethered to the skin or an intradermal plaque. These initial manifestations enlarge over a period of days to weeks, ulcerating in the centre and causing large disfiguring skin ulcers which can sometimes affect the bone, causing permanent disfigurement and long-term disability. Even with effective antimicrobial therapy, BU wound healing is slow, often requiring many months of care ^6^. Studies from Ghana and Nigeria have reported that BU imposes substantial costs on affected individuals, their households ^7, 8^, and the health system ^9^.

Clinical trials in Ghana led the World Health Organization (WHO) to recommend that BU be treated with daily oral rifampicin (10mg/kg dose) and clarithromycin (15mg/kg dose) for eight weeks ^6^. Previous studies have shown that by week 4 of treatment almost 50% of patients have either healed or live MU can no longer be detected by a combined 16S rRNA and qPCR for IS2404. These data highlight that in some patients a short course of antibiotics may be effective ^10^. Preliminary data from Ghana suggests two major factors contribute to variability in healing: the baseline bacterial load and the occurrence of paradoxical reactions ^11^. An additional factor in the variability of healing rates is the possible role of secondary infections of BU wounds. It has been proposed that mycolactone, a toxin produced by MU, may limit occurrence of secondary infections in BU lesions, but supporting evidence is sparse. Several studies conducted in Ghana reported diverse microbial communities being isolated within BU lesions. It remains unclear whether these other bacteria represent true co-infections, secondary colonisation, or opportunistic commensals ^12, 13^. Clinicians sometimes prescribe additional antibiotics to treat potential secondary infections, even though it is unclear whether additional antibiotics are necessary. Such prescribing practices may contribute to antimicrobial resistance (AMR), highlighting the need to understand if and how these isolates interfere with disease resolution ^12^.

Wound dressings with antimicrobial properties may help to improve outcomes in patients with BU. Dialkylcarbamoyl chloride (DACC)-coated dressings irreversibly bind bacteria on wound surfaces resulting in their removal when dressings are changed ^14– 16^. They represent an innovative approach to prevention of secondary infection without use of antimicrobials. A systematic review of seventeen studies, including 3,408 patients with chronic wounds, used silver-impregnated dressings as an active comparator ^17^. A single randomised controlled trial (RCT) and two cohort studies demonstrated that DACC-coated dressings reduced bacterial load to a greater extent than silver-impregnated dressings and one RCT demonstrated a positive effect on wound size reduction ^14, 17, 18^.

Higher doses of appropriate antimicrobials may also further improve outcomes in patients with BU. The 10mg/kg dose of rifampicin, which WHO recommends for BU treatment, was selected because it is the dose used in tuberculosis treatment. Multiple tuberculosis studies have demonstrated that higher-dose rifampicin is safe and well tolerated ^19, 20^ and comparative trials of its efficacy for tuberculosis are underway ^19, 21, 22^. Preliminary data for BU suggest that higher-dose rifampicin may improve outcomes and may facilitate shortening of regimens in part because it may clear viable MU more quickly ^23^. Collectively, these data suggest that high-dose rifampicin is safe and may be an efficacious strategy to improve outcomes for individuals with BU; however, its use has not been evaluated in a clinical trial.

To inform decisions about whether to include DACC dressings and high-dose rifampicin in revised BU treatment guidelines, policy makers require evidence on safety and efficacy, as well as costs and cost-effectiveness. An individual DACC dressing or high dose of rifampicin is more expensive than a standard dressing or rifampicin dose. Investing in these novel interventions may, however, generate cost-savings for households and the health service if they are successful in decreasing time to wound healing, the number of treatment visits, and long-term disability. It is therefore unclear whether DACC dressings and high-dose rifampicin would cost more or less overall than standard treatment. If they prove more expensive, policy makers will want to consider whether the additional costs are worth the health improvements, taking into account the many potential alternative uses of limited resources for healthcare in Ghana. While previous studies have assessed the economic burden of BU treatment on affected individuals ^7, 8, 24^ and the health system ^9, 25^, no analyses have compared the cost-effectiveness of alternative BU treatment regimens.

To address these research gaps, we designed an RCT to investigate whether combining high-dose rifampicin and DACC dressings may significantly improve the time to clearance of viable mycobacteria from wounds, reduce paradoxical reactions and secondary infections, and result in improved outcomes for individuals with BU, as compared with standard-dose rifampicin and DACC dressings. Alongside this trial, we will also conduct a full economic evaluation, in which we will compare the financial and economic costs and outcomes of these two BU treatment strategies from a societal perspective. We will model incremental costs and cost-effectiveness of these strategies relative to one another and to standard care, and explore equity implications of adopting a new strategy.

## Methods

This manuscript has been prepared according to the Standard Protocol Items: Recommendations for Interventional Trials ^26, 27^ (SPIRIT) statement (Additional file 1, *Extended data* ^28^). We report here Protocol version 1.0 dated 23 March 2021.

### Trial design

This is an individually randomised multi-centre trial of high-dose rifampicin and DACC dressing, compared to standard-dose rifampicin and DACC dressings. Participants enrolled in the study will be allocated 1:1 to one of the two treatment groups and followed up until 12 months from the point of randomisation. Consent and baseline data collection will precede randomisation. A summary of the study pathway is shown in Table 1.

**Table 1.** Schedule of procedures for study participants. BU: Buruli ulcer.

Activity	Screening	Week 0	Week 2	Week 4	Week 6	Week 8	Monthly	12 Months	Treatment failure
Written informed consent	X								
Inclusion and exclusion criteria and socio-demographics	X	X							
Randomisation		X							
History and physical exams		X	X	X	X	X	X	X	X
Clinical assessment	X	X	X	X	X	X	X	X	X
Adverse events			X	X	X	X	X	X	X
Clinical assessment of the patient and lesion		X	X	X	X	X	X	X	X
Measurement of lesion size		X	X	X	X	X	X	X	X
Photograph of lesion by digital camera	X	X	X	X	X	X	X	X	X
Counselling and testing for pregnancy	X			X		X			
Swab/FNA for PCR	X	X							X
Swab/FNA for 16S rRNA (0, 2, 4, 6, 8 weeks and then monthly if not healed)		X	X	X	X	X	X		X
VAS		X	X	X	X	X	X	X	X
Functional limitations assessment		X	X	X	X	X	X	X	X
Health-related quality of life (QALY, DALY) for economic evaluation		X	X	X	X	X	X	X	X
Household costs of BU and of care seeking		X	X	X	X	X	X	X	
Socioeconomic status		X	X	X					

### Study setting

The trial is set in BU treatment centres within selected districts in Ghana: Ga West Municipal Hospital, Pakro Health Centre and Wassa Akropong Municipal Hospital. These districts are endemic for BU and the facilities have extensive experience in providing care for the disease. 

### Eligibility criteria

1. Individuals aged five to 80 years
2. Individuals with a nodule, plaque or ulcer, with or without associated oedema
3. Positive test for MU by PCR for IS2404 (identification of MU DNA from a swab or FNA sample)
4. Able to give informed consent (and/or assent) or responsible adult able to give informed consent
5. Able and willing to follow the protocol requirements

### Exclusion criteria

1. Participating in any other interventional study at the time of randomisation
2. Known allergy to any component of DACC dressing materials
3. Known contraindication to either rifampicin or clarithromycin
4. Pregnancy

### Interventions

***Intervention description.*** Individuals who have consented to participate will be randomised to either of the two arms of intervention. In the intervention group (HR+DACC), participants will receive daily high-dose oral rifampicin (20mg/kg) and oral clarithromycin (15mg/kg) for four weeks and DACC-coated dressing applied to the wound and changed every 48 hours until the lesion heals.

The control group (SR+DACC) will receive WHO-recommended daily oral rifampicin (10mg/kg) and oral clarithromycin (15mg/kg) for eight weeks and DACC-coated dressing applied to the wound and changed every 48 hours until lesion heals.

***Criteria for discontinuing or modifying allocated interventions.*** In the event that a participant becomes pregnant after starting the study medication, she will be withdrawn and followed up by an obstetrician throughout the pregnancy to assess any effect on the pregnancy outcome. If a live baby results, then the baby will be followed up by a paediatrician for two years.

Participants on anti-hypertensive drugs will have their blood pressure closely monitored to allow for appropriate decision-making. As part of the trial team, an experienced physician will be available to make decisions regarding treatment, which may include adjustment to doses of antihypertensives if need be.

***Strategies to improve adherence to interventions.*** Community-based surveillance volunteers (CBSVs) within the study districts will be used to support patient follow-up and adherence to trial medications and dressings. Transportation cost of participants to travel between their homes and the health facilities will be reimbursed.

***Relevant concomitant care permitted or prohibited during intervention period.*** All concomitant medication taken by the participant during the study will be monitored and recorded in the Case Report Form (CRF) with trade name and generic name, route or formulation, dosing scheme, the indication, and start and stop dates of administration.

### Outcomes

***Primary outcome.*** The primary outcome measure is the mean time to clearance of viable MU.

***Secondary outcomes***

The following secondary outcomes will be assessed:

1. The proportion of participants with no viable MU organisms, as assessed by a combined 16s rRNA reverse transcriptase/IS2404 real-time qPCR assay, detectable at Weeks 2, 4, and 6, during antimicrobial intervention and at Week 8 after both groups have completed rifampicin and clarithromycin treatment.
2. The proportion of participants achieving complete healing of lesions at Week 20, where complete healing is defined as re-epithelialisation of ulcerated area with stable scar formation during a three-month observation period.
3. The proportion of participants with reoccurrence of BU in each treatment arm; indicator of reoccurrence being the appearance of a new lesion within 12 months of treatment and is not considered as a paradoxical reaction. This will be assessed by the presence of viable micro-organisms detected by culture or 16S rRNA assay after completion of antibiotic treatment and complete healing of the initial lesion.
4. The proportion of participants with paradoxical reaction in each arm of intervention. Paradoxical reaction is defined as:
  a. an initial improvement in the clinical appearance of lesion upon antimicrobial treatment of BU followed by deterioration of the lesion or its surrounding tissues
  b. or the appearance of a new lesion(s)
  c. or examination of excised tissue from the clinical lesion showing evidence of an intense inflammatory reaction consistent with a paradoxical reaction.
5. The proportion of participants who experience secondary infection defined as the occurrence of new purulent discharge within the BU lesion accompanied by microbiological evidence of infection by an organism other than MU.

***Sample size.*** The sample size is based on existing data which demonstrated a mean time to clearance of viable MU with standard dose rifampicin and Vaseline gauze dressings (standard care) of eight weeks ^10^, with a standard deviation of four weeks. Assuming a lower standard deviation of three weeks for high-dose rifampicin (intervention), 100 participants (randomised 1:1) would enable us to detect a reduction of two weeks in mean time to RNA clearance with 80% power and 5% significance. In addition, 100 participants would also allow us to see an increase of approximately 27% in the proportion of patients with healed wounds at 20 weeks in the high-dose rifampicin arm. This is based on a conservative assumption that 50% of wounds would be healed at 20 weeks with DACC dressings and the standard dose rifampicin ^10^. Assuming a loss to follow-up rate of 10%, we therefore plan to recruit 112 participants to ensure that at least 100 participants are followed up for a minimum of 20 weeks.

***Recruitment.*** Recruitment started in November 2021 and continues. The estimated primary study completion date is May 2024 with an enrolment capacity of five participants per month. There will be facility-based recruitment of individuals who seek treatment for BU following counselling and consent. Participants will be recruited from three hospitals in Ghana: Pakro Health Centre, Ga West Municipal Hospital and Wassa Akropong Government Hospital.

### Randomisation, allocation, and blinding

A computer-generated random allocation sequence was prepared at the London School of Hygiene & Tropical Medicine (LSHTM) using block randomisation stratified by site, BU stage (Stage I-III) and specific randomisation number placed in sealed opaque envelopes. This was done in a secure manner and access restricted to avoid any possibility of viewing the allocation sequence. Participants enrolled in the study will be allocated 1:1 to one of the two treatment groups: HR+DACC or SR+DACC. The recruiting clinician will be given randomly generated treatment allocations within sealed opaque envelopes. Once an individual has consented to participate in the trial and has satisfied all the inclusion criteria, an envelope corresponding to their study-specific stratification and number will be opened. The participant will then be offered the allocated treatment regimen with a unique study code, kept with the local site team and the study coordinators. A skilled wound assessor blinded to the allocation will evaluate wound images for assessment of healing to avoid bias and maximise validity of results. Participants at each site will be recruited by the recruiting clinician. It will not be possible to blind participants and clinical research staff administering drugs. Clinical and laboratory data will be collected by staff blinded to the study arm.

### Data collection

***Data collection method and assessment of outcomes.*** At baseline, we will collect demographic, clinical, cost, and health-related quality of life data and photograph lesions. Counselling and pregnancy test will be done for all females of reproductive age at screening (see Table 1). Data on pain assessment using the Visual Analogue Scale (VAS) will be collected fortnightly following randomisation at each scheduled visit.

***Participant timelines.*** Each participant will have 15 scheduled review visits at Week 2, 4, 6, 8 and then monthly until one year following initiation of antimicrobial treatment ( Table 1). Individuals will be followed for a minimum of 20 weeks. Visits beyond 20 weeks will be conducted within the framework of routine care.

***Wound photography and size measurement.*** Wound diameter and area will be recorded by digital photography and measured by computer planimetry using a Silhouette Wound Imaging System (ARANZ, Wellington, New Zealand) at each clinical visit. The Silhouette camera captures an image of the wound. A tracing of the wound boundary will be generated and the wound dimensions will be automatically calculated. These measurements will enable calculation of healing rate at Week 4 and predicted healing time in relation to lesion size and type. The mean diameter, area, depth and volume of the wound will be measured. The same measurements will be made for non-ulcerated lesions (nodule, plaque and oedema).

***MU viability assessment.*** Samples will be taken before and during treatment to assess persistence of viable MU. Swabs will be taken from the undermined edges of ulcerated lesions or FNA samples from the centre of non-ulcerative (nodule, plaque and oedema) lesions at Weeks 2, 4, 6, 8, 12 and 16 (if lesions are not healed) for the combined MU 16S rRNA reverse transcriptase/IS2404 real-time qPCR ^10, 11^.

***Secondary infection and bacterial culture.*** Wounds will be assessed clinically for secondary infection at each time point. In the context of new purulent discharge, a single swab shall be taken for microbiological assessment. Swabs will be plated onto agar and cultured for the identification of potential secondarily infecting bacteria. Organisms will be identified and antibiotic sensitivity testing performed using standard microbiological protocols.

***Resource use and cost data.*** At every scheduled visit, data will be collected on costs incurred by the household that can be attributed to a householder having BU and/or seeking treatment for BU. At Week 0, all costs incurred since onset of BU symptoms will be collected. At each follow-up visit, costs related to BU since the last visit will be collected. These include costs of medicines, hospitalisation, consultation, transportation as well as opportunity costs relating to having and treating BU (for example, missed work or school). Socioeconomic data on household income, expenditure (frequent and non-frequent items) and asset ownership will also be collected at Weeks 0, 2, and 4 to establish a month of income and expenditure data based on prospective data collection with a maximum of 2 weeks’ recall. Information on how households cope with the burden of BU will also be collected at each follow-up visit.

The programme-level costs of resources used in implementing the new interventions (high-dose rifampicin and DACC dressings) are expected to include costs of training health workers, intervention planning, and implementation. These costs will be collected from the project’s activity and financial records; the value of donated items will be estimated based on market prices. Where necessary, government sources will also be consulted for relevant cost data.

***Health-related quality of life.*** Health-related quality of life data will be collected at every scheduled visit ( Table 1) using the EQ-5D-5L instrument developed and validated for Ghana by the EuroQol research foundation. The tool is a brief self-reported preference-based measure which focuses on five dimensions (mobility, self-care, usual activities, pain/discomfort and anxiety/depression) each with five levels of functioning ( *e.g.* no pain-1, slight pain-2, moderate pain-3, severe pain and extreme pain). In addition, we will collect data on patient’s emotional state, experience of pain or itch, and any physical deformity to inform assignment of weights for disability-adjusted life-years (DALYs).

***Monitoring.*** An external Good Clinical Practice (GCP) monitor will be appointed to monitor all aspects of the study, with respect to current GCP, to ensure compliance with government regulations. Prior to the start of the study, the investigator will be informed of the frequency of monitoring visits and will be given reasonable notification before each visit by other designated monitoring bodies.

### Data management

Each participant will be allocated a unique participant identification number which will be used on all electronic documents and photographs. Data will be collected by researchers onto the REDCap platform hosted on a secure server at the School of Medical Sciences (SMS) of the Kwame Nkrumah University of Science and Technology (KNUST). Lesion photographs taken using the ARANZ device will be saved to the secure Silhouette Central SQL server. Data will be encrypted and access will be password-restricted.

***Study documentation and data safety.*** All data collected will be confidential and stored according to GCP standards. Study members with direct access to the data will take all necessary precautions to maintain confidentiality. The study databases will be password-protected and access to password will be authorised by the Principal Investigators. Electronic data files will be stored on operated and dedicated servers at KNUST.

Privacy of the study participants will be maintained by assigning study participants a unique participant identification number. All data, samples and laboratory results will be recorded and analysed with only this unique identification number and no personal identifiers will be used. Analyses of data will be performed on copies of the original data files always ensuring raw data accessibility.

### Statistical analysis

***Statistical methods.*** Statistical analysis of outcomes will be carried out blind to treatment allocation. The primary and secondary efficacy outcome analysis will be carried out using a modified intention-to-treat (ITT) population of individuals who were PCR-positive for BU at baseline. An analysis on a per-protocol population will also be carried out. Analysis will follow a pre-specified statistical analysis plan.

***Populations to be analysed.*** Analyses will be carried out using two different types of dataset: modified ITT and per-protocol.

**The modified ITT dataset**

The modified ITT population will consist of all patients who were PCR positive for BU at baseline.

**The per-protocol dataset**

A per-protocol analysis will be carried out in addition to the primary ITT analysis. The per-protocol dataset will include all individuals who (i) completed the intended course of antimicrobial treatment; (ii) received appropriate wound care; (iii) did not report any major protocol violations; and (iv) did not take any prohibited medications during the study.

***Primary outcome.*** For the primary outcome of mean time to clearance of viable MU, we will use regression models with a Gaussian link function to estimate the mean difference between the two arms. This will be reported with a 95% confidence interval.

***Secondary outcomes.*** For secondary outcomes, appropriate models will be used to examine the effect of the intervention; for example, modified Poisson regression models will be used to estimate risk ratios for binary outcomes. Measures of effects will be reported with 95% confidence intervals. If there is evidence of non-normality in the continuous outcomes, then non-parametric bootstrapping will be used to estimate bias-corrected 95% confidence intervals.

### Economic evaluation

The incremental costs, cost-effectiveness, and equity of using HR and DACC-coated wound dressings relative to alternative strategies will be assessed from provider, household, and societal ( *i.e.*, combined provider and household) perspectives, consistent with reference case specifications ^29^, including the reference case for Ghana if finalized by the end of the study. We will assess actual practice in the trial and develop a model to explore how costs and effects may be expected to vary outside the trial setting in a “real-world” context. The model will combine trial data with additional data sources to inform decisions regarding a potential switch in policy to a treatment strategy incorporating high-dose rifampicin and DACC-coated dressings. The within-trial analysis will be based on the ITT dataset and will compare the costs and outcomes associated with HR+DACC and SR+DACC interventions. In the model we develop, we will project findings over a longer time horizon, if appropriate, and will also include a third comparator, SR plus standard gauze dressings, which reflects current practice recommended by WHO. Health outcomes associated with this current practice strategy will be modelled based on secondary literature and health-related quality of life estimates associated with specific clinical outcomes estimated within our trial; costs will be modelled based on resource use information in the literature ^7, 9^ and unit costs estimated within our trial.

Results of the cost-effectiveness analysis will include mean costs and effects (with distributions for each) under each strategy compared. If one strategy is both more costly and more effective than another, the incremental cost-effectiveness ratio will be presented and compared against relevant cost-effectiveness thresholds ^30, 31^. Deterministic and probabilistic sensitivity analyses will be conducted to explore the sensitivity or robustness of findings to uncertainty and heterogeneity in key parameter values. Results will be presented on the cost-effectiveness plane and using cost-effectiveness acceptability curves. Where more than two alternative strategies are compared, the cost-effectiveness frontier and expansion pathway will be identified ^32^.

Both financial costs, which reflect actual monies paid, and economic costs, which reflect the full value of resources used will be assessed. All costs pertaining to research activities ( *i.e.*, data collection which does not affect outcomes) will be excluded. We will include both the recurrent costs associated with each participant treated, and also the “start-up costs” incurred by providers in switching from current practice to HR and DACC dressings.

The “effectiveness” in the cost-effectiveness analysis will be assessed using quality-adjusted life-years (QALYs) and disability-adjusted life-years (DALYs). Both combine measures of length and quality of life into a single metric, which allows the results of economic evaluations to be compared across different health areas. For QALYs, utility scores between 0 (dead) and 1 (Perfect health) ^33^ will be generated for each participant at each time point by combining population value sets with health states collected with the EQ-5D-5L. If a population value set for Ghana is available by the time of analysis, it will be used; otherwise, we will explore if and how findings may vary when value sets for other African countries ( *e.g.* Uganda ^34^, Ethiopia ^35^) are used. For DALYs, disability weights between 1 (death) and 0 (Perfect health) will be assigned to each participant at each time point by using their responses to patient experience questions (designed for this purpose) to map them onto one of the six skin-related disability states (3 levels, with or without pain or itch) ^36^. For each health state, the standard disability weight (mean and distribution) will be applied. Area under the curve methods ^37^ will be applied to generate total QALYs gained and total DALYs lost for each participant.

Equity dimensions will be examined by assessing variations in incremental costs and benefits of the interventions across socioeconomic and/or demographic groups. 

### Missing data

Every effort will be made to obtain required data at each scheduled evaluation from all participants who have been randomised. Any missing data will be identified and attempts made to obtain until received, confirmed as not available, or the trial is at analysis. Only a small volume of missing data is anticipated and it is therefore unlikely that this will need to be accounted for in any analysis.

However, if necessary we will consider using inverse probability weighting or multiple imputation if missing data were larger than expected and/or there was differential attrition between the trial arms. We would also attempt to ensure that the reason for the differential attrition was fully understood.

### Trial governance

The trial will be overseen by a Trial Steering Committee (TSC). The TSC will have an independent chair and members with clinical expertise. Meetings will be scheduled before enrolment and regular meetings held either by teleconference or face-to-face.

Day-to-day management of the study will be co-ordinated by a central Trial Management Group (TMG) at the Kumasi Centre for Collaborative Research (KCCR) in Tropical Medicine including the Principal Investigator, Senior Trial Clinician and Trial Coordinator and the Trial site Coordinators. This group will provide regular supervision of the trial and ensure that it is conducted in accordance with the principles of GCP and other relevant regulations.

An independent Data Monitoring Committee (DMC) has been established to provide oversight of the study. The DMC will review safety and efficacy data during the active phase of the trial. They will advise on the continued recruitment of trial participants. This committee consists of an independent chair, a statistician and members with clinical and methodological expertise. An independent clinical expert panel (separate from the DMC) has been established to provide external clinical assessment of outcomes where required, for example judging potential paradoxical reactions.

***Auditing.*** The study may be subject to audit by the London School of Hygiene & Tropical Medicine (LSHTM) under their remit as sponsor and other regulatory bodies to ensure adherence to GCP.

***Harms.*** The study has made provision once any Adverse Events (AEs) and/or Serious Adverse Events (SAEs) are identified. An AE/SAE form will be completed and submitted to the study coordination centre with as much detail of the event that is available at that time. SAEs and Serious Adverse Reactions (SARs) will be reported to the study coordination centre within 24 hours of the local site being made aware of the event. Participants will be offered appropriate treatment within the health facilities and followed up or referred to a higher level of care if required. If awaiting further details, a follow-up SAE report will be submitted promptly upon receipt of the outstanding information. The PI will record the event with an assessment of seriousness, causality and expectedness.

Any events relating to a pre-existing condition or any planned hospitalisations for elective treatment of a pre-existing condition do not need reporting as SAEs.

All SAEs assigned by the principal investigator or delegate as both suspected to be related to treatment with investigational product (IMP) and unexpected will be classified as suspected unexpected serious adverse reactions (SUSARs) and will be subject to expedited reporting to the Regulatory Authority. The Sponsor (or delegate) will inform the relevant bodies of SUSARs within the required expedited reporting timescales.

### Ethics

The clinical trial will be carried out in line with the principles of the Declaration of Helsinki and as per the Guidelines of the Food and Drugs Authority (FDA), Ghana using the 2016 ICH E6 R2 Guidelines. Appropriate insurance is in place to cover any harms occurring as a result of trial participation. The trial will ensure all participants are additionally covered by Ghana Health System national insurance policy.

***Consent or assent.*** A trained researcher in Good Clinical Practice (GCP) will screen all individuals for eligibility. Eligible individuals will be provided with a Participant Information Leaflet and verbal information as necessary about the study by a research fellow in English or in another language ( *e.g.* Twi) if preferred by the participant. Written informed consent will be obtained from the potential study participant. For potential study participants under 18 years of age, consent from a responsible adult (parent, relative, guardian, legal representative) will be obtained. Assent shall be obtained from study participants between 12 and 17 years. This will be uploaded into the REDCap database.

***Research ethics approval.*** This study has been approved by the Food and Drugs Authority (FDA) - Ghana (reference number, FDA/CT/2110), the Ghana Health Service (reference number, GHS-ERC 003/01/21), the London School of Health and Tropical Medicine; LSHTM (reference number, 22912) and the KNUST; School of Medical Sciences, Committee on Health Research, Publication and Ethics (reference number CHRPE/AP/030/21). The study has also been registered on the Pan African Clinical Trials Repository with the reference number; PACTR202011867644311. Should there be any changes in the approved documents, the aforementioned authorities will be consulted.

***Plans for communicating important protocol amendments to relevant parties ( *e.g.* trial participants, ethical committees).*** Any protocol amendment will be reported to the Trial Steering Committee for approval. The Sponsor, London School of Hygiene & Tropical Medicine, relevant ethics committees and regulatory authority (see research ethics approval) will subsequently be notified.

***Declaration of interests.*** The principal investigators together with the study team wish to state that they have no conflicts of interest in the trial proposed.

***Access to data.*** Data will be available upon request to the appropriate authorities and data management teams included in the study.

***Dissemination policy.*** Results will be provided to the Ethics Committee and the Regulatory bodies. Results will additionally be disseminated by publication in open access peer-reviewed journals and presented at the annual WHO meetings on BU. For journal publications, authors will be included in line with an overarching publication policy covering the SHARP project and the recommendations of the International Committee of Medical Journal Editors (ICJME).

## Discussion

Neglected tropical diseases, including BU, continue to pose challenges to public health especially in low-and middle-income countries. The WHO continues to advocate for improved diagnosis and management to reduce disabilities associated with NTDs ^38^. While there has been significant progress in the management of BU disease, the median time to wound-healing is still long, with the possibility of affected individuals suffering multiple complications including long term disability as well as a substantial economic burden. In this regard, interventions that reduce healing time and improve treatment outcomes will be a step in the right direction.

Beyond their clinical benefits, such interventions have the potential to create substantial financial and economic savings. Previous studies have shown that the longer healing/treatment time result in higher costs for patients and their caregivers and families. While the cost of antimicrobial treatment is free in BU endemic countries, individuals may incur significant costs related to transportation, subsistence, wound dressings, admission fees, and adjunctive surgery like wound debridement or skin grafting. Further, there might be indirect costs emanating from loss of productive work hours for affected persons and their caregivers. BU patients and caregivers also face significant mental health burden and a reduced quality of life ^4, 39, 40^.

This trial sets out to test the efficacy of an innovative intervention, HR, that has the potential to shorten time to healing and reduce paradoxical reactions and secondary infections. The trial has several strengths: this is the first randomised controlled trial of HR for BU treatment and includes what is expected to be the first cost effectiveness analysis of BU, which is important for ensuring that the limited resources available are used as efficiently as possible to improve outcomes for affected individuals. It is recognised that participants may experience recall bias of the costs of BU prior to the study; however, the cost and health-related quality of life estimates from this trial will add to the limited evidence on this aspect of BU. It is expected that the intervention will improve the clinical outcome of BU-affected individuals while reducing the economic consequences of suffering the condition. The intervention aligns with the objectives of the WHO roadmap for NTDs and has the potential to change WHO recommendations for the treatment of BU in endemic countries.

## Data Availability

**No data are associated with this article.** Zenodo: Additional files for BuruliRifDACC, https://doi.org/10.5281/zenodo.7263508 ^28^ This project contains the following extended data: BuruliRifDACC_ICF.pdf Zenodo: SPIRT checklist for “Buruli-RifDACC: Evaluation of the efficacy and cost-effectiveness of high-dose versus standard-dose rifampicin on outcomes in *Mycobacterium ulcerans* disease, a protocol for a randomised controlled trial in Ghana”, https://doi.org/10.5281/zenodo.7263508 ^28^ Data are available under the terms of the Creative Commons Attribution 4.0 International license (CC-BY 4.0).
