# Supplementary material for: Buruli-RifDACC: Evaluation of the efficacy and cost-effectiveness of high-dose versus standard-dose rifampicin on outcomes in Mycobacterium ulcerans disease, a protocol for a randomised controlled trial in Ghana
Source: NIHR Open Res. 2023 Feb 24;2:59. Originally published 2022 Nov 29. [Version 2] doi: 10.3310/nihropenres.13332.2 (PMC7614217; doi:10.3310/nihropenres.13332.2)
Supplement: Peer review history

# Reviewer report: Reviewer response for version 2

**DOI:** 10.3310/nihropenres.14490.r29034

**Published:** 2023-02-27

**Authors:** Caroline Demangel

**Affiliations:** 1 Immunobiology and Therapy Research Unit, Institut Pasteur, INSERM U1224, Université de Paris Cité, Paris, France

Approved

Is the study design appropriate for the research question?

Yes

Is the rationale for, and objectives of, the study clearly described?

Yes

Are sufficient details of the methods provided to allow replication by others?

Yes

Are the datasets clearly presented in a useable and accessible format?

Yes

Reviewer Expertise:

-

I confirm that I have read this submission and believe that I have an appropriate level of expertise to confirm that it is of an acceptable scientific standard.

# Reviewer report: Reviewer response for version 1

**DOI:** 10.3310/nihropenres.14458.r28953

**Published:** 2023-01-19

**Authors:** Caroline Demangel

**Affiliations:** 1 Immunobiology and Therapy Research Unit, Institut Pasteur, INSERM U1224, Université de Paris Cité, Paris, France

This study aims to improve the current WHO-recommended treatment for Buruli Ulcer. Precisely, the authors propose to test the value of administering a higher dose of antibiotics in combination with Dialkylcarbamoyl chloride (DACC)-coated dressings, whose cost-effectiveness was demonstrated in the care of other wounds. The reasons for testing a higher rifampicin dose is well justified, the methodology is appropriate and clearly described. It includes cost measures alongside efficacy measures, which will be essential to consider changing the WHO recommendations.

 I have only minor comments :

- Page 3: clarify that preliminary data for BU are from mouse studies.
- Page 4: …we have designated RCT...

Is the study design appropriate for the research question?

Yes

Is the rationale for, and objectives of, the study clearly described?

Yes

Are sufficient details of the methods provided to allow replication by others?

Yes

Are the datasets clearly presented in a useable and accessible format?

Yes

Reviewer Expertise:

Buruli ulcer pathogenesiss, Mycobacterium ulcerans biology, Immunology

I confirm that I have read this submission and believe that I have an appropriate level of expertise to confirm that it is of an acceptable scientific standard.

# Response

**Published:** 2023-02-12

**Authors:** Yaw Ampem Amoako

**Affiliations:** Kwame Nkrumah University of Science and Technology, Ghana

Re: “Buruli-RifDACC: Evaluation of the efficacy and cost effectiveness of high-dose versus standard-dose rifampicin on outcomes for Mycobacterium ulcerans disease, a protocol of a randomised controlled trial in Ghana”

 My co-authors and I are grateful to the reviewers for taking time to review our manuscript and for the feedback given. We have taken notice of the comments and have made the suggested changes as detailed below. Our responses are in italics.

**Reviewer 2: Caroline Demangel**

 General comments: This study aims to improve the current WHO-recommended treatment for Buruli Ulcer. Precisely, the authors propose to test the value of administering a higher dose of antibiotics in combination with Dialkylcarbamoyl chloride (DACC)-coated dressings, whose cost-effectiveness was demonstrated in the care of other wounds. The reasons for testing a higher rifampicin dose is well justified, the methodology is appropriate and clearly described. It includes cost measures alongside efficacy measures, which will be essential to consider changing the WHO recommendations.

 I have only minor comments:

 Page 3: clarify that preliminary data for BU are from mouse studies.

*Response: Done. The section now reads  ‘’Preliminary data from mouse studies for BU suggest that higher-dose rifampicin may improve outcomes and may facilitate shortening of regimens in part because it may clear viable MU more quickly ^23^’’.*

 Page 4: …we have designated RCT...

*Response: Done. We have inserted the word ‘’have’’ as suggested. The section now reads: ‘’To address these research gaps, we have designed an RCT to investigate whether combining high-dose rifampicin and DACC dressings may significantly improve the mean time to clearance of viable mycobacteria from wounds, reduce paradoxical reactions and secondary infections, and result in improved outcomes for individuals with BU, as compared with standard-dose rifampicin and DACC dressings.’’*

# Reviewer report: Reviewer response for version 1

**DOI:** 10.3310/nihropenres.14458.r28891

**Published:** 2022-12-21

**Authors:** Lance Waller

**Affiliations:** 1 Department of Biostatistics and Bioinformatics, The Rollins School of Public Health, Emory University, Atlanta, GA, USA

Comments on *NIHR Open Research *manuscript “Buruli-RifDACC: Evaluation of the efficacy and cost-effectiveness of high-dose versus standard-dose rifampicin on outcomes for *Mycobacterium ulcerans* disease, a protocol of a randomised controlled trial in Ghana”.

**General Comments**

1. Many thanks to the authors for a clear description of a carefully designed clinical trial.

**Specific Comments**

1. Page 3, lines 5-6 of right column: The phrasing “patients have either healed or live MU can no longer be detected” is a bit awkward and difficult to parse on first reading. I suggest modifying to “either (a) patients have healed, or (b) live MU can no longer be detected”. Placing “either” before “patients” made the either-or comparison a bit easier for me to digest.
2. Page 4, bottom of left column: I suggest clarifying the primary outcome here. The current text lists three areas of interest but does not mention the primary outcome of interest (mean time to clearance).
3. Page 4, Table 1: Will household costs of BU and of care seeking be examined under treatment failure (the table suggests these will not be considered in this case).
4. Page 6: The proposed effect size for the sample size calculations is somewhat large (25% reduction in mean time to clearance). I suspect there are logistical concerns regarding the number of anticipated cases, but some discussion of anticipated background incidence during the study period would be helpful.
5. Page 6, line 5 of right column: “and pregnancy test” to “and a pregnancy test”.
6. Page 7, under “Primary outcome” on right column: “Gaussian link function” should be “Gaussian error and identity link function” (“Gaussian” refers to the error, not the link function).

Is the study design appropriate for the research question?

Yes

Is the rationale for, and objectives of, the study clearly described?

Yes

Are sufficient details of the methods provided to allow replication by others?

Yes

Are the datasets clearly presented in a useable and accessible format?

Yes

Reviewer Expertise:

Biostatistics, Disease Ecology

I confirm that I have read this submission and believe that I have an appropriate level of expertise to confirm that it is of an acceptable scientific standard.

# Response

**Published:** 2023-02-12

**Authors:** Yaw Ampem Amoako

**Affiliations:** Kwame Nkrumah University of Science and Technology, Ghana

Re: “Buruli-RifDACC: Evaluation of the efficacy and cost effectiveness of high-dose versus standard-dose rifampicin on outcomes for Mycobacterium ulcerans disease, a protocol of a randomised controlled trial in Ghana”

 My co-authors and I are grateful to the reviewers for taking time to review our manuscript and for the feedback given. We have taken notice of the comments and have made the suggested changes as detailed below. Our responses are in italics..

**Reviewer 1: Lance Waller**

 General Comments

 Many thanks to the authors for a clear description of a carefully designed clinical trial.

 Specific Comments

 1. Page 3, lines 5-6 of right column: The phrasing “patients have either healed or live MU can no longer be detected” is a bit awkward and difficult to parse on first reading. I suggest modifying to “either (a) patients have healed, or (b) live MU can no longer be detected”. Placing “either” before “patients” made the either-or comparison a bit easier for me to digest.

*Response: Done*

 2. Page 4, bottom of left column: I suggest clarifying the primary outcome here. The current text lists three areas of interest but does not mention the primary outcome of interest (mean time to clearance).

*Response: Done*

 3. Page 4, Table 1: Will household costs of BU and of care seeking be examined under treatment failure (the table suggests these will not be considered in this case).

*Response: Data on household costs of BU and of care seeking will be collected for all patients up to month 12. No further cost data will be collected after 12 months.*

 4. Page 6: The proposed effect size for the sample size calculations is somewhat large (25% reduction in mean time to clearance). I suspect there are logistical concerns regarding the number of anticipated cases, but some discussion of anticipated background incidence during the study period would be helpful.

*Response: We agree the effect size is large but as the treatment including DACC dressings are more expensive we envisage a significant increase in effectiveness will be needed for the new regime to be cost effective.*

 5. Page 6, line 5 of right column: “and pregnancy test” to “and a pregnancy test”.

*Response: Done*

 6. Page 7, under “Primary outcome” on right column: “Gaussian link function” should be “Gaussian error and identity link function” (“Gaussian” refers to the error, not the link function).

*Response: Done*

 Yours Sincerely,

 Yaw Ampem Amoako

 Corresponding Author
